# Microbiota-gut-brain-axis: a new target of acupuncture therapy for post-stroke cognitive impairment

**DOI:** 10.3389/fmicb.2025.1425054

**Published:** 2025-04-24

**Authors:** Te Ba, Rui Niu, Jianjun Gao, Wenfang Li, Mengwei Dong, Jingjing Duan, Yinan Gong, Sinuo Wu, Zhongxi Lyu, Yang Liu, Ningcen Li

**Affiliations:** ^1^Shanxi University of Traditional Chinese Medicine, Jinzhong, Shanxi, China; ^2^Inner Mongolia Medical University, Hohhot, China; ^3^Research Center of Experimental Acupuncture Science, Tianjin University of Traditional Chinese Medicine, Tianjin, China

**Keywords:** acupuncture, post-stroke cognitive impairment, traditional Chinese medicine, gut microbiota, microbiota-gut-brain-axis

## Abstract

Stroke-induced cognitive impairment is a common complication and an important risk factor for disability. Prevention and treatment of secondary stroke injuries are crucial. Modern research has found that the gut microenvironment can directly or indirectly affect neurological function and cerebral ischemic outcomes, and their crosstalk is achieved through the microbiota-gut-brain-axis (MGBA). Acupuncture, as a promising non-drug treatment, has been recommended for improving post-stroke cognitive impairment (PSCI). However, in recent years, few studies have systematically analyzed the potential mechanisms in this field, and whether acupuncture can improve PSCI through the MGBA remains to be explored. This review comprehensively summarizes literature and shows that, acupuncture, as an adjuvant therapy can play a potential important role in the treatment of PSCI by regulating the microbiota-gut-brain-axis. Acupuncture can repair intestinal epithelial barrier, regulate gut microbiota and serum metabolites, alleviate gut inflammation and neuroinflammation, and regulating HPA axis function, etc. From the studies we have included, the evidence for its effectiveness remains limited, these results should be interpreted with caution due to the low quality of evidence. Future high-quality clinical and experimental studies are needed. This review also discussed the development prospects of acupuncture in improving PSCI via the MGBA, such as genomics, personalized therapy, establishment of standards, and combination therapy, etc. providing new research ideas and scientific and reliable evidence for the application of acupuncture in PSCI.

## 1 Introduction

Intra cerebral stroke, also known as cerebrovascular accident (CVA), is divided into ischemic stroke (IS), intracerebral hemorrhage (ICH), subarachnoid hemorrhage (SAH), and stroke of undetermined type. Stroke is the second largest cause of death and disability in the world after ischemic heart disease (GBD 2019 Stroke Collaborators, 2021). Post-stroke cognitive impairment (PSCI) refers to a series of syndromes that meet the diagnostic criteria for cognitive impairment within 6 months after stroke. Although different subtypes of stroke have different pathogenesis and clinical manifestations, they all have a certain probability of leading to the occurrence of PSCI. The severity of PSCI varies from mild to severe, with up to 60% of stroke survivors experiencing this condition within the first year after stroke (Donnellan and Werring, 2020; Rost et al., 2022). Although there is currently no gold standard for cognitive screening after stroke, several brief cognitive screening tests (≤30 min) have been used to identify PSCI, such as Mini Mental State Examination (MMSE) and Montreal Cognitive Assessment (MoCA). However, since most screenings are not developed for the heterogeneity of PSCI, stroke defects and individual factors of patients may also limit the practicality of screening tools (Kwon et al., 2020). PSCI has the characteristics of low cure rate, slow recovery effect and rapid changes in the condition. The impact of cognitive impairment on daily living activities sometimes far exceeds that of physical impairment. If not actively intervened, it will seriously affect the daily life of patients and cause a heavy burden on families. Cognitive impairment ranks as one of the leading causes of disability worldwide and contributes significantly to the global burden of disease that requires urgent attention in national health policy (Rost et al., 2022). Due to the limitation of treatment time window, the treatment for PSCI patients is currently very limited. At present, the main treatment method is recommended to give priority to rehabilitation training, with drug assisted treatment (such as antihypertensive drugs, lipid regulating drugs, drugs for treating diabetes, antiplatelet drugs and anticoagulants according to the specific conditions of patients), but the economic cost is high, and most wage earners are difficult to support treatment (Huang et al., 2022). At the same time, drugs also have a certain degree of side effects, such as the development of drug resistance, as well as adverse reactions to gastrointestinal symptoms, neurological and psychiatric symptoms, and other systemic symptoms. It is significant to exploring safe and effective treatment methods to alleviate symptoms of PSCI.

Research has found that in addition to symptoms of neurological and cognitive impairment, gastrointestinal dysfunction is also common in PSCI patients. With the gradual improvement of the brain-gut-microbiome theory, more scholars are paying attention to the connection between the brain and the intestine. The gut microbiota and its metabolites have been proven to be closely related to the occurrence and development of central nervous system (CNS) diseases, especially in stroke patients (Han et al., 2023). Researchers have found significant differences in gut microbiota and metabolites between patients with and without PSCI (Feng et al., 2021). Therefore, it is possible that a better intestinal microenvironment may play an important role in maintaining health, and researchers are gradually realizing that treating PSCI can be achieved through benign regulation of the gut microenvironment. In terms of comprehensive treatment of PSCI, acupuncture, one of the oldest and most widely used traditional Chinese medicine technology in the world, has significant advantages in better biological safety and socio-economic benefits. Acupuncture involves the insertion of metal needles into specific points of the body to modulate the meridians, enhance the circulation of *qi* and blood, thus restoring the balance of *yin* and *yang*, and ultimately achieving the goal of disease prevention and treatment (Kaptchuk, 2002). In recent years, more and more clinical trials show that acupuncture can not only promote the recovery of neurological function after stroke, but also have significant benefits for patients with secondary cognitive impairment, which can improve the quality of life of patients with PSCI (Han et al., 2024; Hung et al., 2019). Acupuncture has a multi-target effect, which can simultaneously regulate multiple neurobiological targets and improve brain function. It can also be personalized based on the patient’s specific condition for selecting acupoints and developing treatment plans. Meanwhile, compared to medication treatment, acupuncture has relatively fewer side effects. Acupuncture can regulate the changes of intestinal microbiota, which may be one of the important mechanisms of acupuncture treatment for PSCI, and is also a potential research direction (Zhang Q. et al., 2023). However, in recent years, there have been few studies that systematically analyze the potential mechanisms in this field. This review systematically summarized the relationship between intestinal microenvironment and PSCI, and the role and potential mechanism of acupuncture in preventing and treating PSCI by regulating the intestinal microenvironment, trying to evaluate the role of intestinal microenvironment in the prevention and treatment of PSCI by acupuncture and its possible mechanism, so as to provide new ideas and targets for the research of traditional Chinese medicine in treating PSCI.

## 2 The relationship between intestinal microenvironment and PSCI

The intestinal microenvironment is the most complex part of the human microbiota, composed of gut microbiota, intestinal mucosa, and gut immune system, which are strongly associated with various types of diseases in the human body (Cryan et al., 2020). The microbiota-gut-brain-axis (MGBA) is a bidirectional communication network between gut microbes and brain. The dysfunction of MGBA induced by metabolic disorder is related to stroke and the development of common stroke risk factors such as hypertension, obesity and atherosclerosis. Similarly, changes in gut microbiota are also reflected in the response to stroke and may affect recovery after injury (Honarpisheh et al., 2022).

In addition to digestion and absorption, normal intestinal function also has a strong barrier function against harmful microbial organisms and various toxins produced by the intestine which is achieved through the intestinal epithelial barrier (IEB). IEB, one of the largest interfaces between outside and the body’s internal environment, is composed of epithelial cells, goblet cells, Paneth cells, and enterochromaffin cells (Rodríguez-Colman et al., 2017). Epithelial cells are connected through tight junctions, gap junctions, adhesive junctions and desmosomes, which prevent toxic macromolecules and microbial organisms from passing through and play an innate defense role (Hansson, 2019). Stroke can disrupt the integrity of IEB, leading to damage to the intestinal villous epithelium, increased permeability, damage to intestinal tight junctions, decreased mucus, and decreased defense ability, ultimately leading to an imbalance of microorganisms. Harmful substances cross the intestinal epithelial barrier and enter the extraintestinal organs, increasing the risk of infection after stroke and affecting prognosis (Lee et al., 2023).

Microbial organisms are distributed on the surface and mucous membranes of the human body, with the most abundant part being the gastrointestinal (GI) tract. The gut microbiota consists of approximately 10^14^ cells, including prokaryotes, eukaryotes, viruses and bacteriophages, which are crucial for host metabolism, immune, and nervous system development (Cryan et al., 2019). PSCI can alter the composition of gut microbiota. A comprehensive RNA sequencing analysis was conducted on differentially expressed genes in the ischemic cerebral cortex of mouse brains at pre-stroke and post-stroke day 1 and day 3. It is found that neurodegenerative pathway is the key enriched pathway. And through research on gut microbiota transplantation, it has been demonstrated that the gut microbe-dependent trimethylamine-N-oxide (TMAO) pathway can impact stroke severity (including infarct size and long-term cognitive outcomes) and induce different mRNA expression profiles (Kaur et al., 2023). Similarly, gut microbiota can also influence stroke outcomes through a bidirectional brain-gut axis. A study has found that dysphagia is associated with poor stroke prognosis, including increased risk of breathing difficulties, malnutrition, and even death, possibly due to oral food intake affecting the oral and gut microbiota of patients undergoing enteral nutrition rehabilitation (Arnold et al., 2016; Katagiri et al., 2019). The gut microbiota can not only affect the brain and behavior of stroke patients, but also affect multiple specific brain activities, such as learning, memory, anxiety, etc. Research has found that compared to non-PSCI patients, PSCI patients have significantly higher levels of gut *Enterobacteriaceae, Proteobacteria*. Furthermore, the similar alterations were also observed at the class, order, family, and genus levels (Ling et al., 2020). It was further found that PSCI mice receiving PSCI patient microbiota presented a higher level of *Enterobacteriaceae*, resulting in more severe intestinal damage and cognitive impairment than mice receiving non-PSCI patient microbiota (Wang et al., 2022b). The oral administration of *Escherichia coli* can also cause memory impairment in mice (Jang et al., 2018). In addition, gut microbiota can also affect the main clinical risk factors of stroke. A comprehensive Mendelian randomization study revealed that the abundance of specific bacterial can influence the risk of stroke subtypes, such as cardioembolic stroke, small vessel stroke and large artery stroke (Meng et al., 2023). Age is also a contributing factor to stroke. It is found that the cognitive impairment caused by transplanting feces from elderly people and aged mice into young mice is significantly more severe than feces from young adults and mice (Lee et al., 2020).

The intestinal immune system plays a crucial role in the progression of PSCI. Intestinal immune cells and their secreted inflammatory factors can migrate to the brain through peripheral circulation after the IEB is damaged during stroke (Jiang et al., 2023). It is reported that a significant migration of CD45^hi^ immune cells from the gut to the brain and meninges can be observed at 3 days after stroke. And it was found that CD11c^+^ cells in the intestine significantly tend to migrate from the small intestine to the brain and meninges, leading to inflammation after stroke (Brea et al., 2021), and inflammatory dysfunction of stroke can lead to long-term learning and memory impairments (Gulen et al., 2023). Excessive dietary salt can upregulate the expression of TH17 cells in the small intestine of stroke mice, leading to a significant increase in plasma IL-17. It further promotes cognitive impairment by inhibiting Rho kinase dependent phosphorylation of endothelial nitric oxide synthase and reducing the production of nitric oxide in brain endothelial cells (Faraco et al., 2018).

The enteric nervous system (ENS) can control intestinal behavior and interact with the immune and endocrine systems. ENS can not only interact with microbiota, metabolites, and nutrients, but also interact with immune cells and inflammatory factors to jointly maintain intestinal homeostasis (Rao and Gershon, 2016). ENS structure and neurochemistry resemble that of the CNS, the external connections between CNS and ENS include sympathetic and parasympathetic nerve fibers that directly connect from the hindbrain to the gastrointestinal tract. The vagus nerve and spinal sensory neurons terminate at different locations on the intestinal wall, including the muscular layer and mucosal epithelium. The CNS communicates with the intestine through the gut brain axis (Elenkov et al., 2000; Yoo and Mazmanian, 2017). In ENS, neurotransmitters nitric oxide (NO) and vasoactive intestinal peptide (VIP) are considered to play important roles in the maintenance and protection of neurons (Sandgren et al., 2003). Administration of VIP after stroke in rats can enhance neurogenesis and improve neurological function (Yang et al., 2015). More and more evidence suggested that stroke can affect the gut nervous system. It is found that stroke can trigger the release of central and peripheral galactin-3 causing enteric neuronal loss through by regulating the transforming growth factor β-activated kinase 1 (TAK1) or AMP activated kinase (AMPK) (Cheng et al., 2016). *Paenalcaligenes* and *Escherichia coli* can penetrate the brain through the blood and vagus nerve, leading to cognitive impairment (Lee et al., 2020). In addition, in the study of PSCI, the regulation mechanism of microbiota-gut-brain-axis mediated by gut microbiota and its metabolites (such as gut probiotic metabolites) through vagus nerve and hypothalamus has gradually been reported (Lal et al., 2001). After stroke, it can lead to ecological imbalance in the intestines, producing harmful substances, causing changes in inflammatory reactions and secretion of signaling molecules such as short chain fatty acids (SCFAs) and 3-indoleacetic acid, et al. The inflammatory response after stroke is also considered the core pathological process of secondary brain injury. Inflammation may lead to abnormal immune activation, tissue damage, and ultimately functional loss. By increasing the permeability of the gastrointestinal epithelial layer, inflammation can lead to local dysregulation of the epithelial layer and cause metabolites to escape from the intestine and enter the blood (Vinolo et al., 2011). SCFAs can induce gluconeogenesis, reduce inflammation, and directly interact with vagus nerve afferent nerves (De Vadder et al., 2014; Guo et al., 2022). As a key link between the gut and the brain, the vagus nerve can transmit signals related to appetite, inflammation, and various processes. SCFAs can affect the blood-brain barrier, neurotransmitter levels, and neurotrophic factors, and directly stimulate the vagus nerve to transmit to the nerves, affecting gut-brain communication (Powley et al., 2019). Various intestinal hormones interact with these incoming fibers and ultimately converge in the nucleus tractus solitaries (NTS) located in the medulla oblongata. NTS acts as a relay station for incoming information to the central automatic network (CAN), which includes key areas such as the hypothalamic paraventricular nucleus, locus coeruleus, brachiocephalic nucleus, and limbic system. It can further regulate the HPA axis that responsible for stress response and the autonomic nervous system (Han et al., 2022). But in patients with cognitive impairment after stroke, there is often a decrease in SCFAs. For example, it is reported that the increase of Fusobacteria in the gut and the deficiency of SCFA metabolites in the gut microbiota are significantly correlated with PSCI (Ling et al., 2020).

The microbiota-gut-brain-axis is an interactive system jointly constructed by the brain, gut and gut microbiota, which suggests a certain correlation between the central nervous system and the gut nervous system in the human body. The above studies confirm that the interaction between the intestinal epithelial barrier (mechanical barrier), mucus layer (chemical barrier), intestinal immune cells and their secretions (immune barrier), and intestinal microbiota (biological barrier) is involved in the occurrence and development of stroke and PSCI. A deep understanding of the intestinal microenvironment and its cross-talk with stroke is of great significance for the treatment and prognosis improvement of PSCI.

## 3 Clinical effects of acupuncture treatment for PSCI

We screened the PubMed, Embase, Web of science and Cochrane Library databases for published studies, from January 1998 to December 2023. The search keywords employed were as follows: [“acupuncture” or “electroacupuncture (EA)” or “transcutaneous acupoint electrical stimulation (TAES)” or “transcutaneous electrical acupoint stimulation (TEAS)”] and [“post stroke cognitive impairment (PSCI)” or “vascular cognitive impairment (VCI)” “vascular dementia (VD)”] and [“gastrointestinal microbiome” or “gut flora” or “gut microbiota” or “intestinal microflora” or “intestinal flora”]. A total of 213 articles were identified. The following inclusion criteria were used for screening of the identified articles: stimulation methods included manual acupuncture (MA), EA, TAES and TEAS, and the main diseases studied included cognitive impairment caused by stroke which are related to microbiota-gut-brain-axis. We employed Excel software to manually select references that met the theme. Among them, lacked abstract full text, unrelated to the theme, reviews or meta-analyses were excluded. Finally, 18 full texts of basic research articles and 13 clinical research articles, meeting the theme, were included.

Up to now, many clinical studies have observed the effects of MA or EA on PSCI (Table 1). MoCA, MMSE, the Modified Barthel Index (MBI), the Self-rating Depression Scale (SDS), Activities of Daily Living Scale (ADL), Hamilton Depression Rating Scale (HAMD), Hamilton Anxiety Rating Scale (HAMA), Pittsburgh Sleep Quality Index (PSQI), Loewenstein Occupational Therapy Cognition Assessment (LOTCA) and dementia quality of life questionnaire (DEMQOL) are the main tools for evaluating the efficacy of acupuncture. In clinical practice, the determination of acupuncture treatment plan includes pre-treatment evaluation, personalized selection of acupoints, patient response during treatment, and education for patients and their families after treatment. The principle of selecting acupoints is determined by clinical doctors based on syndrome differentiation and treatment, local acupoint selection, and meridian selection. The scalp is one of the commonly chosen areas for treatment. There is a corresponding spatial relationship between the distribution of acupoints in the head and the functional areas of the cerebral cortex. Acupuncture at acupoints on the head can regulate blood circulation of brain. For example, after 12 weeks of scalp acupuncture and cognitive training, the MMSE total score, orientation, spatial executive function, LOTCA total score, and language orientation control score were improved in PSCI patients. At the same time, brain derived neurotrophic factor (BDNF) and nerve growth factor (NGF) in the serum were also increased, indicating that acupuncture has neuroprotective effects (Xiong et al., 2020). Some researchers have improved the method of scalp acupuncture. a study recruited 660 patients with PSCI and found that after an 8-week course of scalp acupuncture treatment, interactive dynamic scalp acupuncture (IDSA) not only significantly improved cognitive function, but also reduced depression and anxiety, improving patients’ self-care ability, that is better than simple combination therapy (SCT) and traditional scalp acupuncture (TSA) (Zhang S. H. et al., 2022). In addition, besides to the acupoints used in the scalp acupuncture group, specific acupoints can also be added based on the syndrome differentiation. Compared with drug therapy, such as nimodipine or citicoline, acupuncture therapy can significantly improve the MoCA score of PSCI patients (Wang et al., 2016; Yang et al., 2019; Zhang et al., 2013). Acupoints have specific characteristics. There are studies comparing the effects of acupuncture at different acupoints on VD using clinical scales and cerebral functional imaging. It was found that acupuncture at conventional acupoints plus GV20 can affect the inner temporal system, thalamencephalon system and prefrontal cortical system; plus GV26 has a greater impact on the prefrontal cortex system, and plus HT7 has a greater impact on memory. The simultaneous action of these three acupoints can comprehensively affect multiple aspects of the nervous system related to intellectual activities (Huang et al., 2007). Acupuncture also has a good effect on the hemorheology, blood lipid content, and nail fold microcirculation of PSCI patients (Liu, 2004; Lun et al., 2006). Vascular dementia (VD) is also caused by cerebrovascular disease and is a group of diseases with dementia as the main clinical manifestation. It is similar to PSCI in terms of etiology, clinical manifestations, diagnosis, and evaluation, etc. Acupuncture also has a good improvement effect on VD patients. EA can improve the MoCA score of VD patients. And over time, the MoCA score was improved better without any adverse events (Huang et al., 2021; Shi et al., 2015; Zhao et al., 2009). Some studies have also observed the effect of acupuncture on traditional Chinese medicine syndromes of VD patients, and it was found that acupuncture at *Baihui* (GV20), *Sishencong* (EX-HN1), *Shenting* (GV24), *Danzhong* (CV17), *Zhongwan* (CV12), *Qihai* (CV6), *Xuehai* (SP10), *Zusanli* (ST36) and *Neiguan* (PC6) is more effective in treating excess syndromes, such as Liver-yang hyperactivity or phlegm obstruction of the orifices than deficiency syndromes such as kidney essence deficiency (Shi et al., 2014). Acupuncture can also improve superoxide dismutase (SOD) activity in erythrocytes, and lipid peroxide (LPO) level in plasma of VD patients (Gao et al., 2001). It is found that the functional connectivity between the posterior cingulate gyrus-left middle frontal gyrus and the posterior cingulate gyrus-right superior temporal gyrus is positively correlated with cognitive function. Functional connectivity analysis of fMRI showed that after EA, the default mode network function in brain regions such as the posterior cingulate gyrus, left middle frontal gyrus, left anterior cingulate gyrus, left and right superior temporal gyrus, right insula, left precentral gyrus and other brain regions were significantly increased. EA can increase the functional connectivity between the posterior cingulate gyrus and other gyrus in VD patients (Lin et al., 2024). Sum up, acupuncture may be used as a supplementary therapy, further improving the clinical efficacy of PSCI patients.

**TABLE 1:** Clinical effects of acupuncture treatment for PSCI.

References	Acupuncture method	Acupoints	Acupuncture parameters	Outcomes
Xiong et al., 2020	MA	GV20, GV24, GB20, EX-HN1	3∼4h, 3 times a week, 8-week course	MMSE↑, LOTCA↑, FMA↑, mADL↓, BNDF↑, NGF↑
Zhang S. H. et al., 2022	MA	MS6, MS7	30-min, 6 times a week, 8-week course	MMSE↑, MoCA↑, HAMD↓, HAMA, PSQI↓, MBI↑
Zhang et al., 2013	MA/EA	Main acupoints: GV20, GV24, GB20, EX-HN1 TCM syndrome: the kidney essence deficiency syndrome + KI3; phlegm blocking meridian syndrome + ST40; liver-Yang hyperactivity syndrome + LR3; Qi and blood deficiency syndrome + ST36	30-min, 3 times a week, 8-week course	MMSE↑, clock drawing test↓, picture recognition↑
Wang et al., 2016	MA	GV20, EX-HN1, ST2, GB20, GB12, BL10, GV26, HT7, PC6, ST40, SP36, LR3	30-min, 6 times a week, 12-week course	MoCA↑
Yang et al., 2019	MA	ST36, SP10, CV17, CV12, CV6, GV20, GV16, BL15, BL45, HT5, KI6, KI3, GB39, ST40, PC6, BL17	30-min, twice a week (at an interval of 2–4 days), 12-week course	ADAS-cog↓
Huang et al., 2007	MA	LI15, LI11, TE5, LI4, SP10, ST36, SP6, LR3, GV20, GV26, HT7	20-min, 5 times a week, 4-week course	MMSE↑, FAQ↓, Blood Flow↑, glucose metabolism↑, inner temporal system↑, thalamencephalon system activation↑, prefrontal cortical system activation↑, prefrontal cortical system activation↑
Lun et al., 2006	MA + EA	Excess syndrome: LI11, ST40, LR3; Deficiency syndrome: BL18, BL23, ST36	MA: 20-min; EA:30-min, 5 times a week, 9-week course	NO↓, NOS↓
Huang et al., 2021	EA	GV20, GV29, GV24, GV26, GV17, EX-HN1, GB20, HT7, SP6	30-min, 3 times a week, 8-week course	MoCA↑, MMSE↓
Shi et al., 2015	MA	GV20, EX-HN1, GV24, CV17, PC6, CV12, CV6, SP10, ST36, GB20, ST40, LR3, SP6, ST25	30-min, once every other day, 6-week course	MMSE↑, ADL↓
Zhao et al., 2009	EA	EX-HN1, GV20, GV24, GB20	30-min, 5 times a week, 6-week course	MMSE↑, P_300_ (P_3_ latency↓, P_3_ wave amplitude↑)
Shi et al., 2014	MA	Main acupoints: GV20, EX-HN1, GV24, CV17, CV12, CV6, SP10, ST36, PC6 TCM syndrome: the kidney(Shen)-essence deficiency: GB39; phlegm obstruction of the orifices: ST40; blood-letting puncture, for collateral obstruction due to blood stasis: EX-HN11; liver(Gan)-Yang hyperactivity syndrome: LR3; fire hyperactivity: ST44; organ-turbidity retention: ST25; qi and blood deficiency syndrome: CV4	30-min, once every other day, 6-week course	SDSVD↓
Gao et al., 2001	MA	EX-HN1, GV26, PC6, SP6, ST40	15-min, once daily with an interval of 2 days between 2 weeks, 6-week course	HDS↑, SOD↓, LPO↓, P_300_ (P_3_ latency↓, P_3_ wave amplitude↑), Rheoencephalogram↓
Lin et al., 2024	EA	GV20, GV24	30-min, 5 times a week, 8-week course	MoCA↑, STROOP↑

↑, upregulated by acupuncture; ↓, downregulated by acupuncture.

## 4 Acupuncture alleviate PSCI via microbiota-gut-brain-axis

Some clinic and basic studies have shown that acupuncture can alleviate symptoms of stroke and PSCI. It has been proved that acupuncture can effectively regulate intestinal microbiota to treat diseases and keep body in balance. The treatment strategy based on gut microbiota has great clinical potential in acupuncture treatment of PSCI, but its regulatory mechanism is still unclear. Exploring the mechanism of action is crucial for the clinical application of acupuncture in the treatment of PSCI by regulating the gut microbiota. We reviewed and found that acupuncture can improve the symptoms of patients with PSCI by protecting the IEB, remodeling the structure of gut microbiota, inhibiting inflammation, regulating metabolic pathways and regulating the hypothalamic-pituitary-adrenal (HPA) axis (Tables 1, 2).

**TABLE 2:** Effects and mechanisms of acupuncture on microbiota-gut-brain-axis.

References	Acupuncture	Acupoints	Intestinal epithelial barrier	Gut microbiota	Metabolites and metabolic pathways	Immune system	Nervous system	HPA axis
Liu et al., 2023	EA	ST36	ZO-1↓, Occludin↓, Claudin-1↓, MUC2↓	–	–	IL-6↓, IL-1β↓, IFN-γ↓, TNF-α↓	–	–
Wang et al., 2020a	EA	ST36	ZO-1↑, Occludin↑, E-Cadherin↑, MUC2↑, IECs↓	Muribaculaceae↓, Roseburia↓, Faecalibacterim↓, Bifidobacterium↓, Muribaculaceae↓, Roseburia↓, Faecalibacterium↓, Bifidobacterium↓, EscherichiaShigella↑, Erysipelatoclostridium↑	–	MAPK↑(p-ERK1/2↑, p-JNK↑, P-p38↑), TNF-α↓, IL-1β↓, IL-6↓, iNOS↓	–	–
Zhang Q. et al., 2023	EA	ST36, LI4	DAO↑	–	ROS↓, ATP↑, HO-1/PINK1↑, Drp1↓, Mfn1↓, Mfn2↓	IL-1β↓	–	–
Hao et al., 2022	MA	GV20, GV29, ST36	ZO-1↑, Occludin↑, GFAP↓	Bacteroidetes↑, Proteobacteria↓, EscherichiaShigella↓	–	TNF-α↓, LPS↓	–	–
Lv et al., 2022	MA	ST36, SP6, CV4, CV6, GV20	ZO-1↑, Claudin-5↑, Occludin↑	Candidatus↑, Arthromitus↑, Lactobacillus↑, Clostridia_UCG014_unclassified↑, EscherichiaShigella↓, Burkholderia Caballeronia-Paraburkholderia↓, Streptococcus↓	–	IL-1β↓, IL-6↓, TNF-α↓	–	CRH↑, ACTH↓, CORT↑
Zhang Y. et al., 2022	MA	GV20, GV29, ST36	–	Bacteroidetes↑, Proteobacteria↓, Firmicutes↓, EscherichiaShigella↓	–	LPS↓, IL-1β↓, TNF-α↓	–	–
Chen et al., 2022	EA	GV20, GV14, BL23, ST36	–	Elusimicrobia↓	–	IL-1β↓, IL-18↓	–	–
Li S. et al., 2022	EA	GV20, GV14, ST36	–	Akkermansia↑, Lactobacillus↑	IPA↑, UCP2↓, MTR1/PGC-1α↑	–	SOD↑, GSH/GSSG↑, MDA↓, Bcl-2/Bax/Caspase3↓	–
Wang H. et al., 2023	EA	GV26	–	Bacteroidetes↑, norank_f_Bacteroidales_S24-7_group ↑, Bacteroides↑	TC↓, TG↓, HDL↑, LDL↓, ALT↓, AST↓, ALP↓	IL-10↑, IL-6↓, TNF-α↓	–	–
Qiuping et al., 2023	MA	CV17, CV12, CV6, ST36, SP10, SJ5, CV24, CV23, ST7, ST4	–	–	–	Treg↑, Th-17↓, CD4 + RORγt↑, CD4 + Foxp3↓	–	–
Liu et al., 2020	EA	ST36	ZO-1↑, Claudin-1↑	Firmicutes↓	–	CD11b↓, F4/80↓, TLR4↓, MyD88↓, CRP↓, IFN-γ↓	–	–
Wang et al., 2020b	MA	GV20, ST36	–	–	–	miR-93↓, TLR4↓, MyD88↓, NF-κB↓, IL-6↓, TNF-α↓	IL-6↓, TNF-α↓	–
Du et al., 2018	MA	ST36, GV20	–	–	–	TXNIP ↓, NLRP3↓, caspase-1↓, IL-1β↓	–	–
Cao et al., 2021	MA	GV20, ST36	–	–	–	IL-1β↓, IL-6↓, TNF-α↓, p-JAK2↑, p-STAT3↑	α7nAChR↑	–
Dou et al., 2020	EA	ST25, CV4, ST36, SP6	–	Proteobacteria↑, Firmicutes↓, cyanobacteria↓, Bacteroidetes↓, Lachnospiraceae-NK413↓, staphylococci↓, Enterobacter↑, Stenotrophomonas↑, Helicobacter↑	–	–	PGP9.5↓, ChAT↓, nNOS↓	–
Mengzhu et al., 2022	EA	ST25, ST36	–	Ileibacterium↑, Dorea↑, Candidatus Saccharimonas Dubosiella↑, Lactobacillus↑, Lactobacillales↑, Erysipelotrichales↑	–	–	–	CRF↓
Le et al., 2016	EA	ST36, CV4	–	–	–	–	CRH↓, 5-HT↑, 5-HT1AR↑	ACTH↓, CORT↓,
Sun et al., 2015	EA	ST25, ST36, LR3	–	–	–	–	5-HT↓, CGRP↓, NPY↑	–

↑, upregulated by acupuncture; ↓, downregulated by acupuncture. PSCI, post-stroke cognitive impairment; CVA, cerebrovascular accident; IS, ischemic stroke; ICH, intracerebral hemorrhage; SAH, subarachnoid hemorrhage; MMSE, Mini Mental State Examination; MoCA, Montreal Cognitive Assessment; MGBA, microbiota-gut-brain-axis; IEB, intestinal epithelial barrier; GI, gastrointestinal; TMAO, trimethylamine-N-oxide; ENS, enteric nervous system; NO, nitric oxide; VIP, vasoactive intestinal peptide; TAK1, transforming growth factor β-activated kinase 1; AMPK, AMP activated kinase; HPA, hypothalamic-pituitary-adrenal; AJC, apical junction complexes; TJ, tight junctions; AJ, adherent junctions; ZO-1, Zonula Occludens-1; MUC2, mucin2; IECs, intestinal epithelial cells; EA, electroacupuncture; ROS, reactive oxygen species; ATP, adenosine triphosphate; DAO, diamine oxidase; HO-1, heme oxygenase 1; PINK1, PTEN-induced putative kinase 1; Mfn 1/2, Mitofusin 1/2; Drp1, Dynamin-related protein 1; MA, manual acupuncture; MCAO, middle cerebral artery occlusion; BBB, blood-brain barrier; TLRs, toll like receptors; MyD88, myeloid differentiation factor 88; NF-κ B, Nucler factor kappa B; CRP, C-reactive protein; hsCRP, hypersensitive C reactive protein; DAMPs, damage-associated molecular patterns; PAMPs, pathogen-associated molecular patterns; RORγt, orphan nuclear receptor; Foxp3, forkhead box p3; JAK, intracellular Janus Kinase 2; STAT, Activator of Transcription 3; TXNIP, Thioredoxin-interacting protein; NLRP3, NOD-like receptor protein 3; α7nAChR, Alpha-7 nicotinic acetylcholine receptor; CORT, cortisol; CRH, corticotropin-releasing hormone; ACTH, adrenocorticotropic hormone; GR, glucocorticoid receptors; CRF, corticotropin-releasing factor; 5-HT, 5-hydroxytryptamine; CGRP, calcitonin gene-related peptide; NPY, Neuro-peptide Y; IL-6, Interleukin 6; IL-1β, Interleukin 1β; IL-10, Interleukin 10; IFN-γ, Interferon γ; TNF-α, tumor necrosis factor; MAPK, Mitogen-activated protein kinase; p-ERK1/2 phosphorylation extracellular signal-regulated kinases 1/2, p-JNK, nNOS, neurogenic Nitric Oxide Synthase; ChAT, acetylcholine transferase; PGP9.5, protein gene product 9.5; GFAP, glial fibrillary acidic protein; TC, total cholesterol; TG, triglycerides; HDL, high-density lipoprotein cholesterol; LDL, low-density lipoprotein cholesterol; ALT, alanine transferase; AST, aspartate transferase; ALP, alkaline phosphatase; LOTCA, Loewenstein Occupational Therapy Cognition Assessment; FMA, Fugl-Meyer assessment; mADL, modified Daily Living Scale; BNDF, neurotrophic factor; NGF, nerve growth factor; HAMD, Hamilton Depression Rating Scale; HAMA, Hamilton Anxiety Rating Scale; PSQI, Pittsburgh Sleep Quality Index; MBI, Modified Barthel Index; ADAS-cog, Alzheimer’s disease assessment scale-cognitive subscale; ADL, Daily Living Scale; FAQ, Family Attitude Questionnaire; NOS, Nitric Oxide Synthase; SDSVD, Scale of differentiation of syndromes of vascular dementia; HDS, SOD, superoxide dismutase; LPO, lipid peroxide; STROOP, Stroop color-naming condition. Acupoints: ST36, *Zusanli*; SJ5, *Waiguan*; LI4, *Hegu*; LI11, *Quchi*; GV14, *Juque*; GV16, *Fengfu*; GV20, *Baihui*; GV24, *Shenting*; GV26, *Shuigou*; GV29, *Yintang*; SP5, *Shangqiu*; SP6, *Sanyinjiao*; SP9, Yinlingquan; SP10, *Xuehai*; CV4, *Guanyuan*; CV6, *Qihai*; CV12, *Zhongwan*; CV17, *Danzhong*; ST2, *Sibai*; ST25, *Tianshu*; ST37, *Shangjuxu*; ST39, *Xiajuxu*; ST40, *Fenglong*; ST44, *Neiting* LR3, *Taichong*; ST9, *Renying*; KI6, *Zhaohai*; KI3, *Taixi*; PC6, *Neiguan*; HT5, *Tongli*; HT7, *Shenmen*; GB20, *Fengchi*; GB39, *Xuanzhong*;BL10, *Tianzhu*; BL15, *Xinshu*; BL17, *Geshu*; BL18, *Ganshu*; BL23, *Shenshu*; BL45, *Yixi*; EX-HN1, *Sishencong*; MS6, *Anterior oblique line of vertex-temporal*; MS7, *posterior oblique line of vertex-temporal*; EX-HN11, *Shexia*.

### 4.1 Protecting the intestinal epithelial barrier

Acupuncture can protect the structure and function of the IEB and prevent pathogenic substances from crossing the physical barrier, thus maintaining human health. The function of IEB depends on the apical junction complexes (AJC), composed of tight junctions (TJ), adherent junctions (AJ) and desmosomes, can control the entry of gut microbiota into intestinal connective tissue, prevent antigens from passing through IEB (Alonso et al., 2014). TJ is the apical junction of intestinal epithelial cells (IECs), composed of cytoplasmic attachment proteins, transmembrane proteins, and cytoskeletal proteins. They can play a crucial role in maintaining barrier integrity and participate in intestinal epithelial repair (Krug and Fromm, 2020). Electroacupuncture (EA) at Zusanli (ST36) can upregulation TJ proteins (ZO-1, Occludin, and Claudin-1) and mucin protein (E-Cadherin, MUC2) to reduce the apoptosis and proliferation of IECs and intestinal permeability, further repair the mucus and epithelial barriers (Liu et al., 2023; Wang et al., 2020a). Current evidence suggests that stroke destroys the integrity of the IEB and can lead to enterogenic sepsis (Stanley et al., 2016). EA can improve the mitochondrial dynamic balance mediated by the heme oxygenase 1 (HO-1)/PTEN-induced putative kinase 1 (PINK1) pathway and protect the patient’s intestinal barrier (Zhang Y. et al., 2023). Manual acupuncture (MA) at Baihui (GV20), Yintang (GV29) and ST36 can also effectively alleviate the destruction of the intestinal mucosal barrier, manifested as orderly arrangement of intestinal gaps and narrow connecting gaps (Hao et al., 2022).

### 4.2 Regulating the structure of gut microbiota and metabolic pathways

Acupuncture treatment modulates the species abundance of the gut microbiota. A clinical study shows that EA treatment at GV20 and GV24 can effectively alleviate cognitive impairment and balances gut microbiota ecology in patients with subjective cognitive declines. EA can increase the abundance of beneficial bacteria (such as *Roseburia*, *Ruminococcus UCG-014*, *Ruminococcus UCG-013* and *Bacteroides*), and downregulate harmful bacteria such as *escherichiacoli, e.coli* and *Klebsiella* (Tan et al., 2023). Preclinical studies have shown that MA at ST36, *Sanyinjiao* (SP6), *Guanyuan* (CV4), *Qihai* (CV6) and GV20 can enhance the abundance of *Candidatus Arthromitus*, *Lactobacillus* and *Clostridia_UCG-014_unclassified* and decrease the abundances of *Escherichia-Shigella*, *Burkholderia-Caballeronia-Paraburkholderia* and *Streptococcus* (Lv et al., 2022). MA at GV20 and GV29 can enhance the abundance of *Bacteroidota*, *Firmicutes*, *Proteobacteria*, *Campilobacterota*, and *Actinobacteriota* at the phylum level, decrease *Proteobacteria* and *Escherichia-Shigella*, while the bacterial community was dominated by *Proteobacteria* in the MA group. The study also found that acupuncture combined with oral probiotics has a synergistic effect on enhancing efficacy (Zhang Y. et al., 2022). EA at GV20, GV14, BL23 and ST36 can reduce the relative abundance of harmful bacteria (such as the *Catabacter*, *Robinsoniella* and *Desulfovibrio*), while the relative abundance of *Clostridiales-unclassified* is upregulated in the EA + probiotics group, they both can improve cognitive impairment in vascular dementia mice (Chen et al., 2022).

Acupuncture treatment can also modulate the metabolic pathways. Some studies have shown that gut microbiota and its metabolite TMAO have been proved to be related to atherosclerosis. The increased level of TMAO may reflect the severity of atherosclerosis and carotid stenosis, and induce stroke related cognitive dysfunction. Naochang Tongtiao acupuncture applied at anterior oblique line of vertex-temporal, Zhongwan (CV12), CV4, Tianshu (ST25), ST36, Shangjuxu (ST37) and Xiajuxu (ST39) can decrease plasma level of TMAO and improve the nerve function in stroke patients (Wang et al., 2022a). EA can regulate gut microbiota to activate more indole-3-propionic acid, thereby affecting melatonin receptors and improving symptoms in middle cerebral artery occlusion (MCAO) mice (Li S. et al., 2022). Serum metabolomics analysis showed that acupuncture can regulate the N-methylnicotinamide, β-glycerophosphoric acid, geranyl acetoacetate, serotonin and phenylalanine, tyrosine and tryptophan biosynthesis, taurine and hypotaurine, and beta-alanine metabolic pathways (Lv et al., 2022). Acupoint is one of the key factors of clinical effectiveness of acupuncture. Hypertension is a clinical risk factor for stroke, a clinical study used high-performance liquid chromatography tandem mass spectrometry to compare the targeted metabolic phenotype changes induced by two different acupoint treatments. It was found that only in the active acupoint treatment group, Taichong (LR3), Renying (ST9), Taixi (KI3), and Neiguan (PC6) were selected and the metabolites sucrose, fibrodisaccharides, and hypoxanthine were proven to be the most important targets for active treatment (Yang et al., 2018).

### 4.3 Regulating the gut microbiota and immune system

When the IEB is damaged, the permeability of the IEB increases, lipopolysaccharides and other endotoxins produced by bacteria can activate the peripheral immune system, activate immune cells and related signaling pathways, promote the release of inflammatory factors and cause systemic inflammation, and also promote the migration and infiltration of peripheral immune cells to the brain, leading to central nervous system inflammation through the blood-brain barrier (BBB) (Caspani and Swann, 2019). Stroke can trigger inflammatory response and studies have shown that inflammation may affect synaptic function and exacerbate cognitive impairment. A systematic review and meta-analysis evaluated the levels of inflammatory markers in PSCI patients and found that their inflammation (such as CRP, hs-CRP, MMP9, IL-1β, IL-6, IL-10, TNF-α) in blood increased and was negatively correlated with cognitive function. Interventions targeting inflammation can also improve cognitive function in animals, which also seems to explain the clinical use of complement inhibition and engomod in the treatment of PSCI (Tack et al., 2023). Acupuncture can not only repair the intestinal epithelial barrier, but also repair the blood-brain barrier and inhibit the entry of inflammatory factors into the brain. Acupuncture may also improve cognitive impairment after stroke by inhibiting the activation of inflammation related pathways and the expression of inflammatory factors. The content of some types of gut microbiota (such as Bacteroides) is often positively correlated with MMSE and MOCA scores, and has anti-inflammatory properties (Zhuang et al., 2018). Acupuncture can upregulate the expression of Bacteroides and decrease the systemic inflammatory response (Wang H. et al., 2023). EA at GV20 can regulate Treg/γδ T cell polarization (increased the percentage of Treg in the small intestine while decreasing the rate of γδ T cells) and Th17/Treg balance to improve the intestinal barrier integrity of stroke rats (Qiuping et al., 2023; Wang Y. L. et al., 2023). EA at GV20 can not only regulate Tregs and γδ T cells in the small intestine, but also regulate them in the ischemic brain, inhibit the mobilization of intestinal T cells to brain, which indicated its effect on inhibiting inflammation (Wang Y. et al., 2023). Intestinal epithelial cells contain various pattern recognition receptors, such as toll like receptors (TLRs), which can recognize invading damage-associated molecular patterns (DAMPs) and pathogen-associated molecular patterns (PAMPs), and regulate subsequent inflammatory responses (Wicherska-Pawłowska et al., 2021). EA can alleviate both macroscopic and microscopic colonic inflammation, upregulate the expression of claudin-1 and ZO-1 to repair the intestinal barrier, remold the overall structure of the gut microbiota, and inhibit pro-inflammatory factors IFN-γ, TNF-α, and IL-6 through the MyD88-dependent TLR4 signaling pathway, preventing excessive immune response throughout the body (Liu et al., 2020). MA at ST36 can inhibit TLR4/MyD88/NF-κB signaling pathway mediated by miR-93 in experimental vascular dementia rats to alleviate cognitive impairment associated with inflammation. The expression of inflammatory cytokines (IL-6 and TNF-α) in the hippocampus and plasma were significantly decreased after treatment (Wang et al., 2020b). Thioredoxin-interacting protein (TXNIP) plays an important role in oxidative stress and NOD-like receptor protein 3 (NLRP3) inflammasome activation. MA at ST36 and GV20 can decrease TXNIP, NLRP3, caspase-1, and IL-1β in the hippocampus of vascular dementia rats (Du et al., 2018). The above studies have found that acupuncture can downregulate the expression of inflammatory factors in the gut, serum and brain, regulating the gut microbiota and immune system, improving stroke and cognitive impairment. Alpha-7 nicotinic acetylcholine receptor (α7nAChR) is a ligand gated ion channel expressed in the central and peripheral nervous systems and has been proven a key component to regulate inflammation. MA at these two acupoints can also promote cognitive function by activating α7nAChR and its downstream JAK2-STAT3 pathway, and found that this combination of acupoints is better than GV20 + GV24 and ST36 + SP10 acupoints combination (Cao et al., 2021). A systematic analysis of 42 studies with a total of 1,486 animals also showed that among all acupuncture regimens, GV20 + ST36 combined with 14-day MA had the best improvement effect on cognitive function (Li G. et al., 2022).

### 4.4 Regulating the gut microbiota and nervous system

ENS can act as a local neural mechanism to independently control intestinal behavior and interact with the immune and endocrine systems. Gut microbiota can activate stress circuits in the CNS through the vagus and sensory neurons of ENS (Cryan et al., 2020). NO and VIP are considered to play important roles in maintaining and protecting neurons in ENS (Joly et al., 2021). Stroke can affect the ENS, and acupuncture may improve stroke and subsequent cognitive impairment by regulating gut microbiota and ENS. Studies have found that EA at ST25, ST36, CV4 and SP6 promotes the diversity of intestinal microbiota, significantly improving the pathological ENS score, serum neurotransmitter levels, and intestinal transport rate in mice (Dou et al., 2020). The functional activity of the brain depends on signal transmission between neurons and glial cells, which mainly relies on neurotransmitters (Alkasir et al., 2017). Neurotransmitters are chemical substances that can transmit information between neurons through synapses, thereby controlling behaviors such as movement, emotion, and memory. The neurotransmitter substances produced by gut microbiota are also closely related to the host’s behavior (O’Donnell et al., 2020). In additional, certain bacterial taxa in the intestine can also produce enzymes that catalyze the synthesis of neurotransmitters in the brain. A study has found that neurotransmitters from the intestine act on the brain through blood circulation, local stimulation of the intestinal nervous system, and rapid signal transduction of the vagus nerve (Chen et al., 2021). Another study found that focal cerebral ischemia and global cerebral ischemia have different effects on the survival of intestinal neurons, which may reflect the difference in the immune response of peripheral nerves, also indicating the mechanism of acupuncture application also needs further exploration (Cheng et al., 2018).

### 4.5 Regulating HPA axis

The HPA axis is one of the main neuroendocrine systems that respond to stress, and can produce glucocorticoids, participate in cognitive processes such as neural development, learning and memory. The HPA axis includes cortisol (CORT), the corticotropin-releasing hormone (CRH), and the adrenocorticotropic hormone (ACTH). Research shows that stress can significantly affect microbiota-gut-brain-axis through HPA axis (Rusch et al., 2023). For example, CORT receptors are expressed on epithelial cells, immune cells, and intestinal endocrine cells of the intestine. They can not only directly affect intestinal function, but also affect the gut microbiota by altering intestinal transport time, intestinal permeability, and nutrient availability. Cortisol can affect brain function by binding to glucocorticoid receptors (GR) in the hippocampus, amygdala, and prefrontal cortex of the brain (Carabotti et al., 2015). The imbalance of the HPA axis can affect gut microbiota and further affect post-stroke cognitive function. Acupuncture may improve post-stroke cognitive impairment by regulating the HPA axis. MA at ST36, SP6, CV4, CV6 and GV20 can increase the expression of CRH and CORT and inhibit the expression of ACTH (Lv et al., 2022). EA at ST25 and ST36 can modulate the dysbiosis of gut microbiota and suppress the overexpression of corticotropin-releasing factor (CRF) in colon tissues (Mengzhu et al., 2022). EA at CV4 and ST36 can reduce the expression of CRH mRNA in the hypothalamus, downregulate ACTH and CORT levels in plasma, and significantly increase hippocampal serotonin concentration and 5-hydroxytryptamine receptor 1A (5-HT1AR) mRNA and proteins expression, improving adverse emotions in rats (Le et al., 2016). It is found that neurotransmitter 5-HT can mediate changes in the hypothalamus, spinal cord, and colon, and spinal cord 5-HT and calcitonin gene-related peptide (CGRP) are closely related to behavioral assessment. EA at ST25, ST36 and LR3 can regulate the expression of 5-HT, CGRP, and Neuro-peptide Y (NPY) in the distal colon, spinal cord, and hypothalamus (Sun et al., 2015). The role of acupuncture in regulating the HPA axis has been widely reported. However, limited studies have shown whether acupuncture can improve PSCI by regulating the HPA axis. Currently, most of the evidence is related rather than direct, and further exploration is needed in the future.

## 5 Discussion and conclusion

**FIGURE 1:**
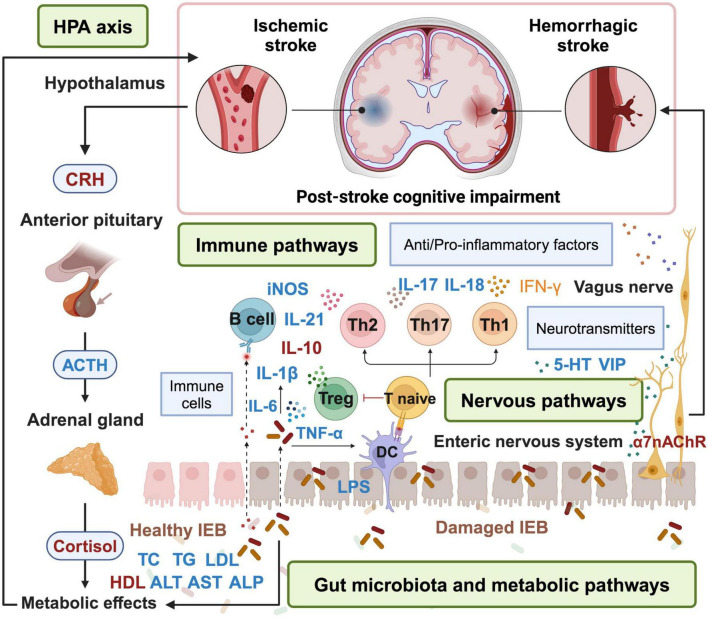
Acupuncture treatment for PSCI via microbiota-gut-brain-axis. Acupuncture can alleviate symptoms of PSCI by repairing the damaged IEB, remodeling the structure of gut microbiota, inhibiting inflammation, regulating the release of neurotransmitters, metabolic pathways and HPA axis. Factors in blue are down-regulated by acupuncture, while factors in red are up-regulated by acupuncture. HPA, hypothalamic-pituitary-adrenal; CRH, corticotropin-releasing hormone; ACTH, adrenocorticotropic hormone; DC, dendritic cell; IEB, intestinal epithelial barrier; VIP, vasoactive intestinal peptide; 5-HT, 5-hydroxytryptamine. The figure is created with BioRender.com.

Prevention and treatment of secondary stroke injuries are crucial. As an important participant in the pathological and physiological events of cognitive impairment in stroke, the structure and function of the gut microenvironment can directly or indirectly affect neurological function and cerebral ischemic outcomes, and their crosstalk is achieved through the microbiota axis. The microbiota-gut-brain axis includes the nervous system (central and enteric nervous systems), endocrine system (HPA axis), and immune system (intestinal and central immune systems), and is an information exchange network connecting the gut and brain. It can transmit information “bottom–up” from the gut to the brain and “top–down” from the brain to the gut bidirectionally. Here are certain correlations between intestinal epithelial barrier, gut microbiota and serum metabolites, gut inflammation, neuroinflammation and HPA axis function. This review demonstrates that, acupuncture, as an adjuvant therapy can play a potential important role in the treatment of PSCI by regulating the microbiota-gut-brain-axis (Figure 1). Acupuncture is a promising non-drug treatment for reducing cognitive symptoms. In the analysis of acupoints included in the literature, it was found that the commonly used local acupoints on the head are *Baihui* (GV20), *Shenting* (GV24), *Shuigou* (GV26), *Yintang* (GV29), and *Fengchi* (GB20) and *Fengfu* (GV16). The commonly used acupoints on the limbs include *Hegu* (LI4), *Waiguan* (SJ5), *Neiguan* (PC6), *Zusanli* (ST36), *Sanyinjiao* (SP6), *Yinlingquan* (SP9), *Xuehai* (Xuehai), *Taixi* (KI3), *Zhaohai* (KI6), and *Taichong* (LR3). The abdominal acupoints include *Guanyuan* (CV4), *Qihai* (CV6), *Zhongwan* (CV12), *Danzhong* (CV17), *Tianshu* (ST25), etc. The acupoints on the head have the functions of clearing the brain, awakening the brain, and enhancing intelligence. In the theoretical system of acupuncture and meridians, the Du meridian plays an important role in the cognitive function of the brain. Acupuncture of head points can protect damaged neurons by affecting glucose metabolism, regulating neurotransmitters, releasing and improving patients’ intelligence, and promoting the improvement of cognitive function. Acupuncture at acupoints in the abdomen and lower limbs can improve cognitive impairment by regulating the balance of gut microbiota, improving the gut microenvironment, promoting the release of beneficial gut microbiota and neuroactive substances, regulating the immune system. We also found that the acupuncture protocols used in clinical trials lack consistency, which may be related to the diversity of acupoint selection and compatibility, heterogeneity of operating parameters and stimulation techniques, diversity of acupuncture methods, and lag in mechanism research and standardization. Traditional Chinese medicine emphasizes “acupoint selection based on syndrome differentiation”, and different studies select acupoint combinations based on syndrome types. However, these acupuncture protocols reflects the advantages of individualized treatment, but weakens the comparability between different studies. The operation of acupuncture also depends on the doctor’s experience, and it is difficult to blind the performer. Only a few trials can blind subjects, evaluators, and statistical analysts. There are various cognitive function assessment tools, including MMSE, MoCA, ADL scales, but the threshold definition and sensitivity are different. Some studies combine neuroimaging (such as fMRI) or biomarkers, but there is a lack of unified standards for detection methods and time points. There is still no international consensus on specific acupoint combinations and stimulation parameters for acupuncture treatment of PSCI. In the future, it is necessary to develop core acupoint combinations and develop layered treatment plans based on syndrome classification. Doctors need to unify operating parameters, standardize electroacupuncture frequency, needle retention time, and treatment course. It is also necessary to optimize the design of sham acupuncture to reduce the interference of comfort effect and promote multi center large sample research. It is expected to improve the quality of clinical evidence for acupuncture treatment of PSCI and promote its international application.

Although studies have shown that acupuncture may affect PSCI through the MGBA, the specific mechanism of action is still not very clear. The MGBA involves complex interactions between multiple systems such as the nervous system, endocrine system, and immune system. Further research is needed on how acupuncture can precisely regulate these systems to improve cognitive impairment. At present, research on acupuncture for treating PSCI through the MGBA is mostly small sample clinical studies or animal experiments, lacking large-scale, multicenter clinical studies to further confirm its effectiveness and safety. In the future, more basic studies and multicenter randomized controlled studies should be conducted to explore whether acupuncture can improve PSCI through microbiota-gut-brain-axis, and determine the causal relationship among them. By utilizing modern biotechnology such as genomics, proteomics, metabolomics, etc., explore the effects of acupuncture on gut microbiota, neurotransmitters, inflammatory factors, and the relationship between these factors and cognitive function improvement. There is no unified standard for the optimal time point, treatment frequency, and duration of acupuncture treatment, which poses certain difficulties for clinical application. Different treatment plans may produce different therapeutic effects, and further exploration is needed to determine the optimal treatment plan. Further clinical treatment standards for acupuncture treatment of PSCI through the MGBA can be developed, including diagnostic criteria, treatment plans, efficacy evaluation, etc., in order to improve the reproducibility and reliability of treatment. Exploring the combined application of acupuncture and other treatment methods is also a research direction that we can consider, such as drug therapy, rehabilitation training, psychological therapy, etc. By integrating multiple treatment methods and exerting synergistic effects, the therapeutic effect can be improved.

In addition, in the literature we have compiled, we found that two different stimuli, MA and EA, were used for PSCI. There may be some differences in the mechanisms of using MA and EA to treat diseases. MA can stimulate acupoints and regulates the circulation of meridians, qi, and blood. EA, on the basis of traditional acupuncture, can enhance the intensity and duration of acupuncture by stimulating acupoints with electric current. Different frequencies and waveforms of current can also produce different therapeutic effects. These two are interrelated, both regulating physiological functions of the human body through stimulation of acupoints to achieve the goal of treating diseases. In the study of acupuncture treatment for PSCI, there is currently no comparison between MA and EA treatment. In our study, we found that the reason why researchers used MA for stimulation may be to eliminate interference from electrical currents. We also searched some other literature and found that in some diseases, researchers compared the therapeutic effects of MA and EA. For example, it was found that EA treatment is not superior to MA treatment for lower back pain, and the two therapies have similar efficacy in reducing pain and disability in chronic non-specific back pain (Comachio et al., 2020). However, due to the relatively limited research in this area, experts have indeed provided us with new research directions that we can further explore in the future. There is also no unified standard for the optimal time point, treatment frequency, and duration of acupuncture treatment, which poses certain difficulties for clinical application. Different treatment plans may produce different therapeutic effects, and further exploration is needed to determine the optimal treatment plan. Further clinical treatment standards for acupuncture treatment of PSCI through the MGBA can be developed, including diagnostic criteria, treatment plans, efficacy evaluation, etc., in order to improve the reproducibility and reliability of treatment. Exploring the combined application of acupuncture and other treatment methods is also a research direction that we can consider, such as drug therapy, rehabilitation training, psychological therapy, etc. By integrating multiple treatment methods and exerting synergistic effects, the therapeutic effect can be improved.
